# Corrigendum to “Risk Factors for Falls and Fragility Fractures in Community-Dwelling Seniors: A One-Year Prospective Study”

**DOI:** 10.1155/2017/3814602

**Published:** 2017-07-02

**Authors:** Sacha Song, Joy C. MacDermid, Ruby Grewal

**Affiliations:** ^1^Schulich School of Medicine & Dentistry, University of Western Ontario, London, ON, Canada N6A 5C1; ^2^University of Windsor, Medical Education Building Room 1100, 401 Sunset Avenue, Windsor, ON, Canada N9B 3P4; ^3^Division of Orthopedic Surgery, University of Western Ontario, Hand and Upper Limb Center, St. Joseph's Health Care, 268 Grosvenor Street, London, ON, Canada N6A 4L6; ^4^School of Rehabilitation Sciences, McMaster University, 1400 Main Street West, Hamilton, ON, Canada L8S 1C7

In the article titled “Risk Factors for Falls and Fragility Fractures in Community-Dwelling Seniors: A One-Year Prospective Study” [1], there was an error regarding the FRAX® tool, which should be clarified as follows.

The article notes: “FRAX is a tool that can help assess fracture risk. Launched by the World Health Organization (WHO) in 2008.” However, the World Health Organization (WHO) did not develop, test, or endorse the FRAX tool or its recommendations [2]. The metabolic bone disease unit at the University of Sheffield that developed FRAX was a WHO Collaborating Centre from 1991 to 2010, but treatment guidelines must undergo a formal process before they can be endorsed by the WHO.
